# The Growth of Reform

**Published:** 1905-07-29

**Authors:** 


### THE GROWTH OF REFORM.

### THE ROYAL SOUTH HANTS AND SOUTHAMPTON HOSPITAL.

Important changes, amounting in many cases to a revolution, in old methods have come with such rapidity in the case of many provincial as well as metropolitan hospitals during the last ten years that their effects have hardly received the attention which they merit. By way of illustration we have invited Miss Mollett, one of the ablest matrons in the country, who has occupied this responsible post at the Royal South Hants and Southampton Hospital for upwards of twelve years, to record the changes and improvements which have taken place there during this period. We publish the first instalment of her interesting report to-day, and shall hope that other hospital matrons may be moved to send a brief account of the developments made in their institutions in recent years.

### TWELVE YEARS' IMPROVEMENTS AND CHANGES.

By Miss M. Mollett.

I have been fortunate enough to hold the post of matron at the Royal South Hants and Southampton Hospital for the last 13 years. During that time it has developed from an inconvenient and old-fashioned country hospital, with an insufficient nursing staff, into a large and well-organised county hospital, with a flourishing training-school for nurses, and the account of our changes may be of some interest to your readers. The last 12 years have been years of great progress and change in county hospitals generally, but only a few matrons have remained in office throughout the period of reconstruction, and have seen it through from the beginning to the end. Before commencing I must describe as briefly as possible the old building and the old conditions as they were when I first came in 1892.

#### What the Hospital was Like in 1892.

The central block of the hospital, which is still used in part as the administrative block, was built in 1843. It is a solid, rather handsome structure—faced, as is the whole of the old part of the hospital, with cement; it was opened in 1844 under the name of the Royal South Hants Infirmary. In 1851 two wards were added, and again in 1867, when the "Crabbe block" was built, containing by far the two best of the old wards. A good out-patient department was built in 1887, whilst in 1890 some cottages adjoining the hospital were converted into a nurses' home. The number of paper beds was 104, the number occupied about 80.

When I came into residence in June 1892, I was struck with the dejected appearance of the building; the cement facing had never been renewed, in parts it was green and cracked badly, in others huge pieces of cement had bodily peeled off; inside the hall was painted a dingy nondescript shade, and the same dreary colouring was repeated in the wards; the theatre or operation-room was situated just under the roof in an attic reached by a winding staircase, so narrow and awkward that it was only by lifting the stretcher over a corner of the bannisters and turning with the utmost care, the patient being nearly perpendicular, that a case could be conveyed to and from operation. The "theatre" was provided with one small sink with doubtful hot water, whilst a very small open fireplace on which one kettle could just be balanced provided for the whole supply of boiling water. Round the theatre were grouped three smaller attics in which slept the night nurses—four, at the most five, for the whole hospital. These attics were badly ventilated and when the theatre was being used it was absolutely impossible for the night nurses to sleep.

The two female wards had no bathrooms at all, and both were badly lighted and ventilated. By a curious architectural arrangement the passage to the larger male and female wards, both medical and surgical, passed through the small wards. Thus, for instance, all the patients, living or dead, going to or from the male medical ward, were carried through the children's ward, as well as all their provisions and appliances. The lavatories, small and cramped, of course not fitted with either sluice pans or convenient sinks, opened straight into the wards. The whole of the basement was surrounded by a deep area, preventing both light and air from entering the basement freely; it was damp and unhealthy. The kitchen range had been fixed in the small scullery, where at one small sink all the washing-up had to be done and all the vegetables cleaned, whilst the scullery-maid underwent a process of slow roasting.

Both the laundry and the mortuary were the most awful places of their kind I had ever seen. The small hand laundry, designed originally for a hospital of about 20 beds, with a couple of nurses, had apparently never been enlarged or altered, and simply bulged with dirty linen on Monday morning, which left its walls on Saturday evening very little the better for its sojourn. At that time there was a rule in force by which the nurses brought down all the foul and soiled sheets and rinsed them through themselves at a sink in the laundry. The mortuary was too small, without any private place for friends to see their dead, without proper arrangements for post-mortem examinations — a plain, neglected, whitewashed building.

The nurses' home of converted cottages was never satisfactory—it was damp, the rooms small and low, the passages without any ventilation. A few years ago, after the death of my assistant matron from diphtheria, it was inspected by the medical officer of health and at once condemned.

The nursing staff was entirely inadequate; a probationer system had just been introduced, but it was quite in its infancy. The total allowance consisted of seven nurses (including the out-patient nurse and the night superintendent) and 12 probationers, 19 in all, for a hospital of 104 beds, with a large proportion of typhoid and severe surgical cases. When I came the number of probationers was eight or nine, I forget which, and for some time I had the greatest difficulty in keeping our number up to 12. The work was very hard and not well organised, the probationers having among other things to commence their day's work by polishing the ward floors. One probationer was in charge of both the medical and surgical female wards at night, which were on different landings, and contained 20 beds each. One night probationer was also in charge of 20 male medical beds and two small children's wards of eight beds each. Under such circumstances it was, of course, a physical impossibility for a nurse to prevent a delirious typhoid or pneumonia patient from getting out of bed. The authorities had tried to counteract the evil of insufficient night nurses by placing an electric bell-push over the bed of every patient, *even the infants' cots*.

There were only three wardmaids in the hospital, and they were also responsible for the cleanliness of the nurses' home, so that neither the wards nor the home were ever properly cleaned.

The ward bedsteads were very low, wide, and fitted with very old-fashioned spring-mattresses in wooden cases. These were a grand nesting-place for bugs, and I have never forgotten the shock I received when a scrubber came to me at the annual ward cleaning for an order "for some of that stuff to kill bugs with." On investigation I found that the ancient wooden bedsteads had been a breeding-place for bugs for years, and that they were regularly decimated, as far as possible, annually at spring cleaning. I am happy to say the committee at once procured new spring iron bedsteads of an excellent and most satisfactory modern type.

The minor ward necessaries were very short. There were no proper drawsheets, very few mackintosh sheets, no water-beds (we had to hire), no dressing cloths or bedpan covers, no bath thermometers, and we were in a chronic condition of being short of enema syringes and clinical thermometers. The grumbling about the diets was never ending, although they were not bad on paper; the supply of hot water was simply farcical, the whole of it (with the exception of the two modern male baths) was drawn from the kitchen boiler; hot-water taps were scattered in profusion all over the establishment, but they were simply there for show; we used cold or boiled it in kettles. From the above it is easy to reconstruct the old hospital, for the whole was in keeping. After you had been in it for a short time you saw that it was obviously a small hospital that had been enlarged without any definite plan, and whose work had grown very considerably without any adequate provision having been made for expansion. There were not even enough hands to keep it clean; economy had been pushed to the point where a hospital acquires the dejected and forlorn look of a "place not properly kept up."

(*To be continued.*)

